# Quantitative trait loci analysis of hormone levels in Arabidopsis roots

**DOI:** 10.1371/journal.pone.0219008

**Published:** 2019-06-28

**Authors:** Sangseok Lee, Lidiya I. Sergeeva, Dick Vreugdenhil

**Affiliations:** 1 Laboratory of Plant Physiology, Wageningen University and Research, Wageningen, The Netherlands; 2 Organic Agriculture Research Institute, Gyeongsangbuk-Do Agricultural Research & Extension Services Centre, Uiseong, Gyeongbuk, South Korea; Iwate University, JAPAN

## Abstract

Quantitative trait loci (QTL) analyses for five groups of hormones, including cytokinins in Arabidopsis roots were performed using recombinant inbred lines (Ler×Cvi). Significant QTLs were detected for cytokinins, jasmonic acid and salicylic acid. Separate analysis of two sub-populations, viz., vegetative and flowering plants revealed that many of the QTLs were development-specific. Using near-isogenic lines, several significant QTLs were confirmed; three co-localized QTL regions were responsible for determining several cytokinin metabolites. Using a knock-out plant, a functional role of zeatin *N*-glucosyltransferase gene (*UGT76C2*) underlying a large-effect QTL for levels of tZ-*N*-glucosides and tZRMP was evaluated in the metabolism of cytokinins. Pleotropic effects of this gene were found for cytokinin levels in both roots and leaves, but significant changes of morphological traits were observed only in roots. Hormone QTL analysis reveals development-specific and organ-dependent aspects of the regulation of plant hormone content and metabolism.

## Introduction

Plant hormones are naturally occurring organic substances that influence complex processes in plant development at extremely low concentrations. Hormone levels vary during plant development, between organs, e.g., roots and shoots, and between vegetative and flowering stages [1–3]. Within a species, hormone concentrations may also vary between different varieties [2]. Such variations of plant hormones are obviously determined by genes involved in anabolic and catabolic pathways, transport facilitators and/or signalling components [4, 5].

Naturally occurring variations among different accessions for a particular trait are due to allelic diversity and insertion/deletion (INDELs) of bases including gene duplication in genomes in a species [6]. Even allelic difference at a single locus can contribute to pleiotropic differences in growth and fitness [7]. Such allelic variations may induce changes in metabolic profiles, as shown by Chayut *et al*. [8]; allelic variation of the melon’s Or gene (*CmOr*) resulted in an increase of beta-carotene accumulation in melon fruit. Hence, the quantitative variation of plant hormones within a species is also expected to be due to genetic heterogeneity.

The quantitative variation of hormone levels can be considered as a complex phenotypic trait, determined by multiple loci, which may interact with each other. Quantitative trait loci (QTL) analysis has been used to identify genomic regions responsible for polygenic traits. In comparison with direct-mutagenesis approach, QLT analysis is more likely to identify genes encoding regulatory proteins or rate-determining enzymes, which will reveal important biochemical targets [9].

Natural genetic variation controlling various traits has intensively been studied using recombinant inbred lines (RILs) in plants, especially in the model species *Arabidopsis thaliana* [10, 11]. Several genes regulating glucosinolate contents have been elucidated through linkage mapping in Arabidopsis [12, 13]. Except two studies about loci related to levels of abscisic acid (ABA) in maize and salicylic acid (SA) in Arabidopsis [14, 15], to our knowledge, there are at present no QTL-based data yet on genetic elements determining hormone contents in Arabidopsis or other plant species, despite the crucial roles of hormones in plant growth and development.

In this study, we investigated if, and to what extent, a quantitative genetic approach based on hormone levels in roots, may reveal genes involved in regulatory or metabolic pathways. We chose a RIL population, derived from the parental lines Landsberg erecta (Ler-0) and Cape Verde Island (Cvi-0), since these lines showed divergent traits for root hormone contents in our previous study [16]. Several QTLs for levels of cytokinins (CKs) and jasmonic acid (JA) were confirmed using near-isogenic lines (NILs). Further, by using a loss-of-function mutant of a CK *N-*glucosyltransferase gene situated at a QTL region of CK metabolites, its functional role was evaluated.

The present study shows that genetic elements controlling hormone levels in plants can be unravelled through quantitative genetic analysis, providing a powerful method to understand hormone metabolism.

## Materials and methods

### Plant materials and phenotyping

Ler×Cvi RILs, developed and genotyped by Alonso-Blanco *et al*. [17], were chosen based on our previous study on the natural variation of hormone levels in roots of Arabidopsis accessions [16]. Seeds of 149 lines were placed on wet filter paper in a Petri-dish at 4°C for 4 days in darkness and after that sown on the top of 0.5 mL cylindrical plastic tube, from which the bottom had been cut off, and that was filled with 0.5% agar in half strength of Hoagland's nutrient solution (pH 5.5). Tubes with seedlings of each line were grown in hydroponic containers (70 plants per 10 litres, renewing the nutrient solution once a week). Plants were grown at 21°C during the light period (10 h) and at 18°C during the dark period (14 h). Light intensity and humidity were fixed at 125 μMol m^-2^s^-1^ and 70% respectively.

After 5 weeks of culture, roots were harvested between the 5th and 8th hour within the 10 hours daytime period. For hormone analysis of RILs, NILs and knockout (KO) plants, six to seven roots of each line were pooled for one biological replicate. Pooled roots were immediately ground in liquid nitrogen and freeze-dried for 24 hours. For hormone analysis in leaves (Columbia-0 and KO plants), two largest rosette leaves in each plant were chosen and leaves of four plants were pooled for one biological replicate. On the day of harvest, the developmental stage (vegetative or flowering) and root phenotypic traits of each line were recorded. If any of the replicates within a line showed visible bolting, the line was scored as ‘flowering’. Before phenotyping of root fresh weight, the drops of liquid on roots were removed with paper tissue.

### Hormone extraction and purification

For each RIL, one biological replicate was used to extract endogenous hormones and further analysed for hormone quantification. For parental lines, NILs and KO plants, 4 to 5 biological replicates were used. Powder of lyophilized roots (2.5 mg) was extracted and purified with the same methods used in our previous study [16].

### Quantitative analysis of plant hormones

For IAA, ABA, CKs, JA and SA, all of ultra-pressure liquid chromatography (UPLC)-tandem mass spectrometer methods were the same as those of our previous study [16]. In order to achieve better chromatographic separation of isomers of CK glucoside (e.g., tZ7G, tZ9G and tZOG) in NILs and KO plants, ammonium formate was used as described by Novak *et al*. [18]. Ten microliter of sample was injected onto an Acquity UPLC HSS column (50 x 2.1 mm, 1.8 μm; Waters) and eluted by binary mobile phases, A (15mM ammonium formate, pH 4.0) and B (100% methanol), with a constant flow rate (0.25 ml min^-1^) at 40°C for 14 min. The linear gradient elution was performed as follows: 0~0.01 min, 10% eluent B; 0.01~8.0 min, 10 to 50% eluent B; 8.0~8.5 min, 50 to 100% eluent B; 8.5~9.5 min, 100% eluent B; 9.5~10.5 min, 100 to 10% eluent B. At the end of gradient, the column was equilibrated to initial conditions for 3.5 min. The effluent was introduced in electrospray ion (ESI) source of mass spectrometer with operating parameters: capillary voltage, 3 kV; cone voltage, 22 V; source and desolvation temperature, 150°C and 650°C; cone and desolvation gas flow, 50 and 1000 L hour^-1^; MS mode collision energy, 2 V; MS/MS mode collision energy, 10 V. Two selective transitions were used to perform multiple reaction monitoring (MRM) detections (S1 Table). All data were processed by TargetLynx in MassLynx Software (Version 4.1, Waters, USA). The quantification of each targeted analyte was based on a linear calibration curve that covered the range of concentrations of compounds in samples, and corrected by the recovery rates of the isotope-labelled internal standards.

### QTL analysis

To map QTLs using the RIL population, a set of 99 markers spaced over the Arabidopsis genetic map was selected from the previous published RIL Ler/Cvi map [17]. These markers spanned 482 cM, with an average distance between consecutive markers of 5 cM and the largest genetic distance being 12 cM. QTL analysis was performed using the computer program MapQTL version 6.0 [19] as described by Bentsink *et al*. [20]. Both interval mapping and multiple QTL model (MQM) methods were used to locate QTLs linked to the molecular markers as described in the reference manual. The estimated additive effect and the percentage of variance explained by each QTL affecting a trait, were obtained with MapQTL in the final MQM model. For this, different cofactor markers were tested around the putative QTL positions [19], selecting as final cofactors the closest marker to each QTL. A logarithm of odds (LOD) threshold of 2.5 was applied to declare a significant QTL, which corresponds to a general genome-wide significance of P<0.05, as determined by permutation tests (1,000 repetitions). QTL regions for 95% confidence were determined by 2-LOD support interval that constructs two positions, left and right of the point estimate of the QTL, which have a LOD score of two less than the maximum.

For the purpose of revealing developmental stage-specific QTLs, all RILs were sorted into two groups according to the transition of plants for flowering at day 35, i.e., the day of sampling (the ratio between vegetative and flowering line was, 48.9: 51.1). For each trait in the two groups, QTL regions were independently determined by MQM analysis with automated suggested cofactors.

### QTL confirmation analysis

A selected set of NILs carrying small Cvi introgressions in Ler background, which was developed by Keurentjes *et al*. [21], was tested to confirm some chosen QTLs in the Ler×Cvi RIL population. Significant differences of tested traits were compared between Ler and NILs through analysis of variance (ANOVA, p<0.05).

### PCR to confirm homozygous KO plants

Candidate T-DNA (KO) plants were chosen from TAIR (www.arabidopsis.org) website and obtained from the European Arabidopsis Stock Centre (NASC, UK). The PCR was performed to screen T-DNA insertion and its homozygosity for the gene of interest. Forward and reverse primers were designed on the website, T-DNA Primer Design (http://signal.salk.edu/tdnaprimers.2): for SAIL 801B03 and SAIL 1151A08, forward 5’-TCGAAAAACGTCAACAAAACC-3’, reverse 5’-AGAGTCCTCTGCTTCCGATTC-3’; SALK 102337, forward 5’-GCAGATCATAGGAACCCCTTC3’, reverse 5’- TCCGAACCAAGGGATATCTTC-3’. Reaction mixtures were prepared with reagents: 1 μl, d’NTPs (5mM); 0.4 μl, forward and reverse primer (10 pM); 0.15 μl, Firepol (1U); 7.15 μl, water; 1.2 μl, PCR buffer; 1.5 μl, MgCl (2.5mM); 1.0 μl, DNA. The following conditions were performed to amplify DNA fragments: denaturation at 95°C for 5 min followed by 30 s at 95°C, annealing at 55°C for 30s and extension at 55°C for 2 min, which was cycled 30 times and ended with final amplification at 72°C for 10 min.

## Results

### Variation of hormone levels in roots of Ler×Cvi RIL population

**Fig 1:**
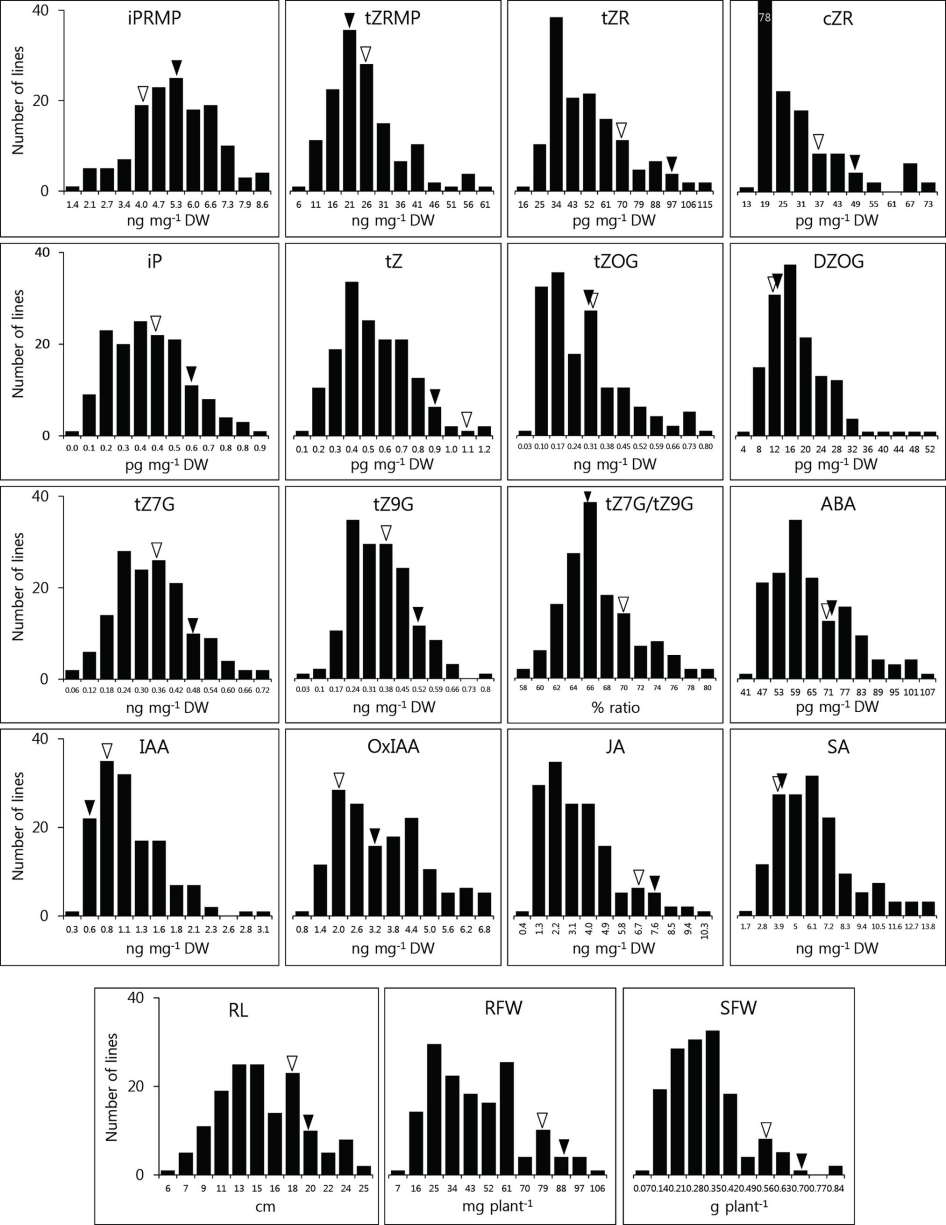
Frequency distributions of root hormone levels and phenotypic traits in the Ler×Cvi RIL population. Arrows indicate levels of hormones in parental lines: Ler, black; Cvi, white. For abbreviations of hormone compounds and phenotypic traits: see legends in Table 1.

To identify the genetic loci affecting endogenous hormone levels in Arabidopsis roots, hormone levels were determined in 35-day-old-roots of a Ler×Cvi RIL population. For most hormones and their metabolites, transgressive segregation was found within the population (Fig 1), the levels ranging 10- to 30-fold between extremes. It indicates that both parental lines carry allelic variants that increase or decrease values of traits. For ABA, only a limited range of concentration variation was observed, viz., three-fold between extremes. In most cases, normal distributions were found, although the distribution ranges were skewed for several compounds, e.g. *cis-*zeatin riboside (cZR) and JA. Weight phenotypic traits showed transgressive segregations below the parental values.

In several cases, significant correlations were found between levels of hormone compounds (Table 1). Among CK metabolites, tZ positively correlated with its two precursor metabolites, tZRMP and tZR. The two CK *N-*glucosides (tZ7G and tZ9G) showed highly significant positive correlations between each other, and both were positively correlated with tZOG. A significantly positive correlation was found between the two CK ribosides (tZR and cZR), and both of them showed similar pattern of correlations to other hormones. 2-oxindole-3-acetic acid (OxIAA) that is known as an irreversible catabolite of IAA [22, 23] negatively correlated with IAA. Low correlation values were observed between ABA and other hormones. Also, some correlations between phenotypic traits and hormones were found. Root fresh weight (RFW) and root length (RL) showed significantly negative correlations with levels of JA, whereas levels of CK glucosides positively correlated with RFW and shoot fresh weight (SFW).

**Table 1:** Correlations between hormone levels and phenotypic traits in roots of 35-day-old plants of the Ler×Cvi RIL population.

Variables	iPRMP	iP	tZRMP	tZR	tZ	tZ7G	tZOG	tZ9G	DZOG	cZR	IAA	OxIAA	ABA	JA	SA
iP	_0.18_														
tZRMP	_0.21_	_0.02_													
tZR	_-0.23*_	_-0.12_	_0.31*_												
tZ	_0.10_	_0.03_	_0.22*_	_0.42*_											
tZ7G	_0.06_	_0.13_	_-0.11_	_-0.06_	_0.16_										
tZOG	_-0.01_	_0.19_	_-0.13_	_-0.12_	_0.10_	_0.22*_									
tZ9G	_0.19_	_0.21_	_-0.14_	_-0.20_	_0.12_	_0.83*_	_0.22*_								
DZOG	_0.10_	_0.17_	_-0.03_	_-0.22*_	_0.07_	_0.38*_	_0.12_	_0.35*_							
cZR	_-0.40*_	_-0.24*_	_-0.04_	_0.57*_	_0.21*_	_0.09_	_-0.10_	_-0.12_	_-0.18_						
IAA	_0.20_	_0.11_	_0.14_	_0.05_	_0.03_	_-0.24*_	_-0.05_	_-0.09_	_-0.01_	_-0.08_					
OxIAA	_-0.19_	_-0.09_	_0.11_	_0.22*_	_0.16_	_0.16_	_-0.02_	_0.00_	_-0.06_	_0.29*_	_-0.33*_				
ABA	_-0.02_	_0.10_	_0.05_	_-0.02_	_-0.03_	_-0.06_	_-0.09_	_-0.06_	_0.01_	_0.10_	_0.19_	_-0.05_			
JA	_-0.13_	_-0.23*_	_-0.03_	_0.32*_	_0.23*_	_0.13_	_-0.07_	_-0.12_	_-0.18_	_0.51*_	_-0.18_	_0.38*_	_-0.04_		
SA	_0.12_	_0.01_	_-0.01_	_0.00_	_-0.09_	_-0.27*_	_0.04_	_-0.16_	_0.00_	_-0.08_	_0.33*_	_-0.23*_	_0.11_	_-0.04_	
RL	_0.21_	_0.04_	_-0.07_	_-0.11_	_0.00_	_-0.05_	_0.21*_	_0.09_	_-0.08_	_-0.18_	_0.15_	_-0.23*_	_-0.08_	_-0.21*_	_0.02_
RFW	_0.19_	_0.20_	_-0.06_	_-0.30*_	_-0.05_	_0.14_	_0.30*_	_0.32*_	_0.04_	_-0.27*_	_-0.03_	_0.05_	_-0.05_	_-0.28*_	_-0.20_
SFW	_0.03_	_0.08_	_-0.07_	_-0.05_	_0.07_	_0.35*_	_0.30*_	_0.39*_	_0.00_	_-0.06_	_-0.15_	_0.31*_	_-0.13_	_0.01_	_-0.29*_

Star marker (*) indicates the significant correlation (p<0.01). Abbreviations for hormone compounds and phenotypic traits are: isopentenyl riboside monophosphate (iPRMP), isopentenyladenine (iP), *trans-*zeatin riboside monophosphate (tZRMP), *trans-*zeatin riboside (tZR), *trans-*zeatin (tZ), *trans-*zeatin-*O*-glucoside (tZOG), *trans-*zeatin-7,9-glucoside (tZ7,9G) dihyro-zeatin-*O*-glucoside (DZOG), *cis-*zeatin riboside (cZR), indole-3-acetic acid (IAA), 2-oxindole-3-acetic acid (OxIAA), abscisic acid (ABA), jasmonic acid (JA), salicylic acid (SA), root length (RL), root fresh weight (RFW) and shoot fresh weight (SFW).

### QTLs for CKs, SA, JA and root phenotypic traits

**Fig 2:**
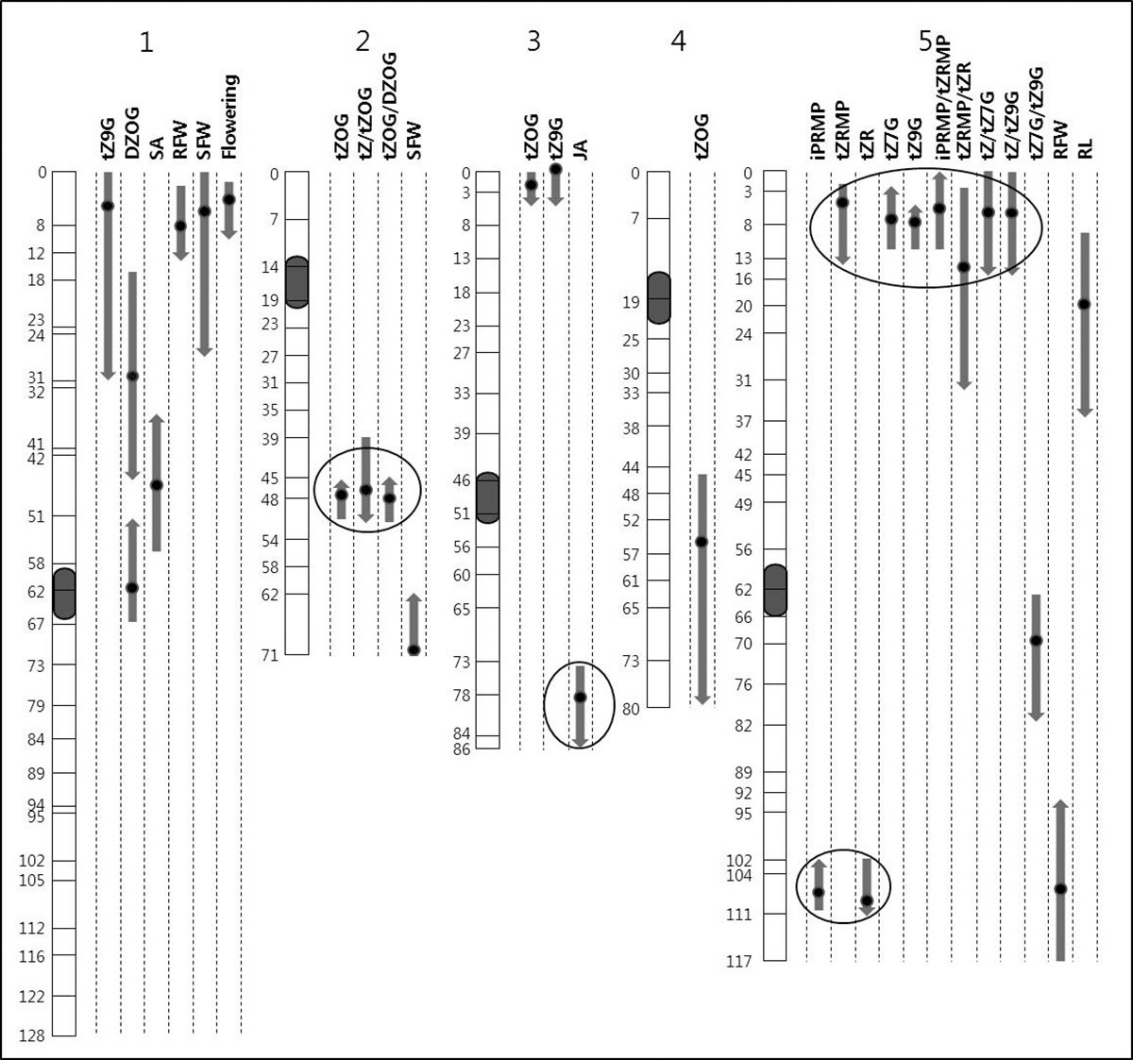
Genetic locations of hormonal and root phenotypic QTLs. Numbered bars represent chromosomes; the numbers along the bars are genetic map position in centi-Morgans. Directions of arrows indicate additive (allelic) effects: upward, Ler alleles increase trait values; downward, Cvi alleles increase trait values. Lengths of arrows indicate 2-LOD intervals (95% confidence). Dots in arrows indicate the highest LOD position for each QTL. Circles indicate four QTL regions that were tested with related NILs for QTL confirmation analysis.

Among five classes of hormone, significant QTLs for CK, JA, SA and for several phenotypic traits were observed (Fig 2, S2 Table). The explained variances of these loci for the various traits ranged from 5% (tZOG on the chromosome 4) up to 25% (tZ9G on the chromosome 5). Twelve QTL regions related to CK metabolites and only one QTL for JA and SA were observed. Within CK metabolites, a few QTLs co-localized, but the directions of additive effects of these were not the same. No significant QTLs were found for IAA, OxIAA and ABA.

QTLs for levels of tZ9G, DZOG and SA were detected at the upper arm of chromosome 1, but with different positions. The QTLs for tZ9G co-localised with those for RFW, SFW and flowering time, and they all showed the same allelic effect. This region coincides with the earlier reported QTLs for flowering time and root growth in the same RIL population [21, 24, 25]. Interestingly, a QTL for tZ9G was found, but no significant QTL for its isomer (tZ7G) was detected at the same region.

Biosynthetic pathways in plant metabolism are composed of a series of precursor-to-product pathway, in which rate-controlling steps (usually enzymatic reactions) are involved [26, 27]. Several cases of such precursor-to-product relation existed within CK metabolites measured in this study. Since most of the conversions are catalysed by enzymes, which are encoded by genes, we expected that QTLs for ratios of metabolic compounds should be also detectable. Indeed, within CK metabolites, six metabolite ratio QTLs were found. Most of these QTLs co-localized with those of single CK compounds except one for tZ7G/tZ9G ratio, which was newly detected at the lower arm of chromosome 5.

### Development stage-dependent QTLs for hormone levels

At the time the samples for hormone analyses were taken (35 day), 51% (76 out of total 149) of the lines were visibly bolting. Therefore, we wondered if QTL analysis could be done for vegetative and flowering lines separately, and to what extent the results would be different from the analysis based on the whole population. Using the same RIL population, Keurentjes *et al*. [28] demonstrated that values of LOD and the number of detected QTLs decreased with decreasing population size and that the degree of the changes differed from trait to trait. In our case, most QTLs detected in the whole population were detectable in the sub-populations, but some of them were no longer significant. Among 23 significant QTLs for hormones and root traits found in the whole population, 13 QTLs appeared to be below the significance threshold (2.5 LOD score) due to lower power in the smaller subsets. Interestingly, some new QTLs were detected in the subsets that had not been detected in the full set of lines.

**Fig 3:**
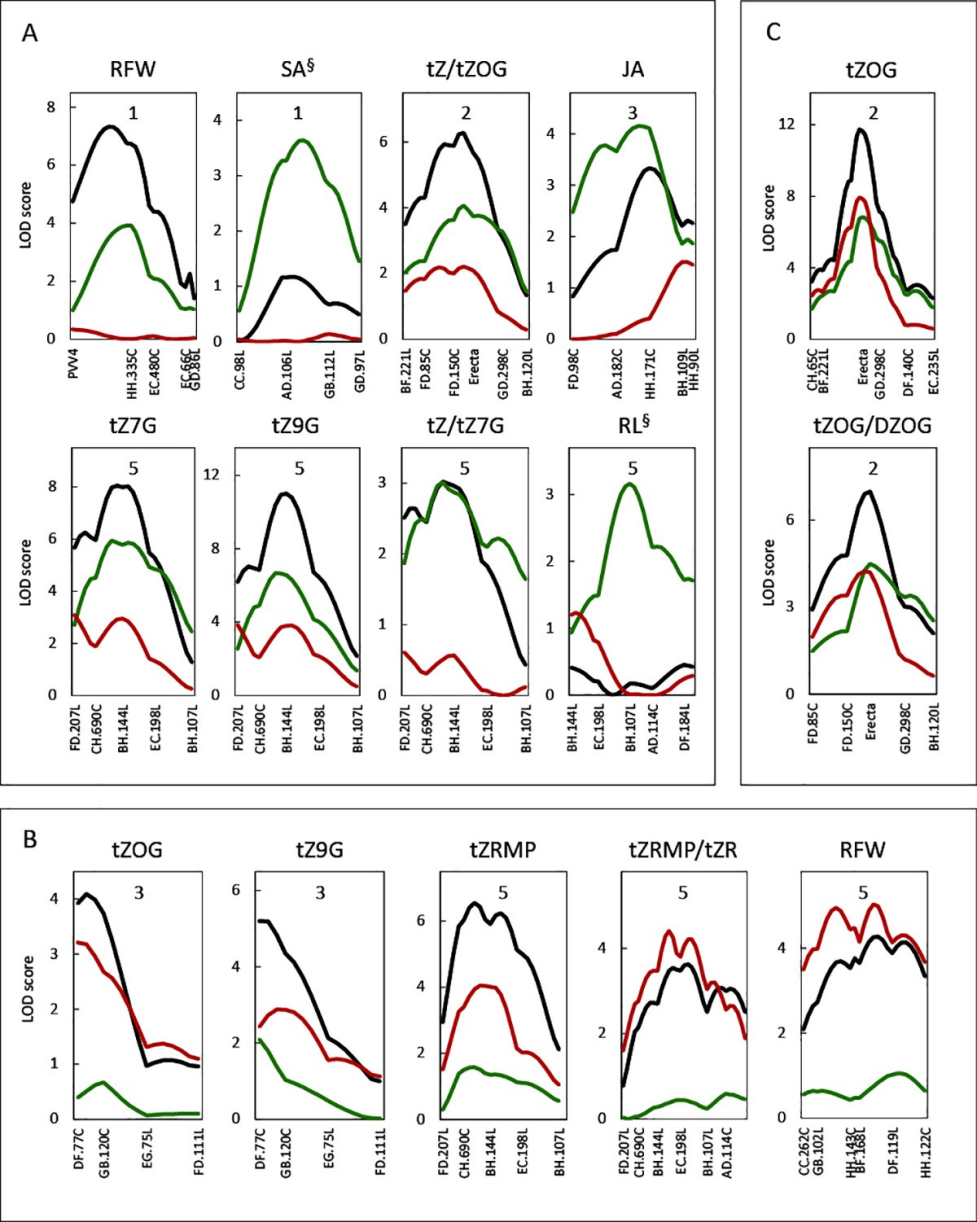
Development-specific QTLs based on vegetative- and flowering lines. (A) vegetative-specific QTLs. (B) flowering-specific QTLs. (C) non-development-specific QTLs. X-axes present genetic markers. Black lines are QTL-profiles when all lines (149) were included, green lines from only vegetative plants, and red lines from only flowering plants. Numbers in the boxes refer to chromosome number. Only the parts of chromosomes containing significant QTLs are depicted.

Fifteen of the QTLs from the sub-sets could be categorized into three classes: vegetative-specific (8 QTLs), flowering-specific (5 QTLs) and non-development-specific (2 QTLs) (Fig 3). Among the 8 QTLs specific for the vegetative stage, two QTLs for SA and RL were newly detected in vegetative lines and had not been found in the whole population (^§^ superscript marked in Fig 3, S2 Table). In some cases, two developmentally distinct QTLs for the same trait were observed: two QLTs for RFW, one for the vegetative stage on the top of chromosome 1, the other specific for the flowering stage on chromosome 5; two QTLs for tZ9G, one for vegetative stage on the top of chromosome 3 and the other for flowering stage on chromosome 5. For the level of tZOG, two QTLs were found, one on the top of chromosome 3 was flowering-specific, and the other on chromosome 2 was non-development-specific. Among four QTLs for metabolite-ratio traits, developmental specificities of three QTLs were the same as those for either precursors or products, e.g., flowering-specific QTLs for the level of tZRMP and tZRMP/tZR ratio on chromosome 5. This implies that the same genetic loci may underlie both QTLs, being functional during the same developmental stage.

### Confirmation analyses of hormone QTLs

Four QTL regions, affecting levels of CK and JA, were further investigated using available NILs, with small Cvi introgressions into Ler background [21].

#### a. QTL for CK metabolites at chromosome 5

**Fig 4:**
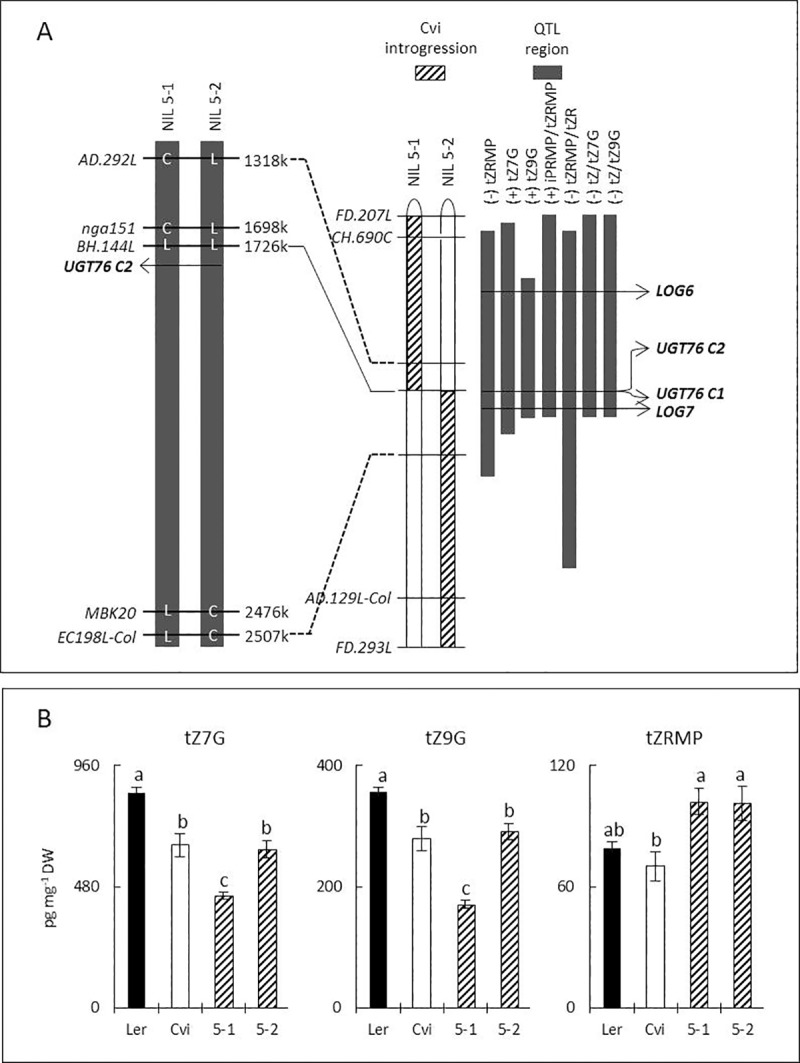
Confirmation of QTLs for levels of tZ7G, tZ9G and tZRMP. (A), genotype of NIL5-1 and NIL5-2. L (Ler-0) and C (Cvi-0) in the black columns on the left indicate allelic identities of parental lines at the markers found in the two NILs. In the middle bars, diagonally-striped areas represent Cvi introgression regions in the NILs. On the right, black columns indicate QTL regions in 2-LOD confidence interval for traits of tZ7G, tZ9G, tZRMP and CK metabolite-ratios. Plus and minus in parentheses in front of trait names indicate additive effects. Horizontal arrows represent positions of known genes related to CK biosynthetic pathways in Arabidopsis: two Lonely Guy (LOG) genes and two CK *N*-glucosyltransferases (CK-*UGT*). (B), compound levels in tested lines, (Ler-0, Cvi-0, NIL5-1 and NIL5-2). Vertical lines on the bars present standard errors and letters on the top of each bar indicate significant differences between lines with a confidence interval of 95% (ANOVA, Duncan).

Maps of two NILs (5–1 and 5–2) and QTL regions for three CK compounds and their metabolite-ratios at the top of chromosome 5 are described in Fig 4A. For tZ7G and tZ9G, significant lower levels of the compounds were observed in both NILs, and NIL5-1 showed significantly lower levels of compounds than NIL5-2 (Fig 4B). It implies that the QTL region may include two different loci, being divided around BH.144L, one in the upper position having a stronger effect than the other in the lower position. Both NILs showed higher levels of tZRMP compared to Ler, which is consistent with the presence of a locus affecting tZRMP level, although the differences were not significant.

#### b. QTL for tZOG at chromosome 2

**Fig 5:**
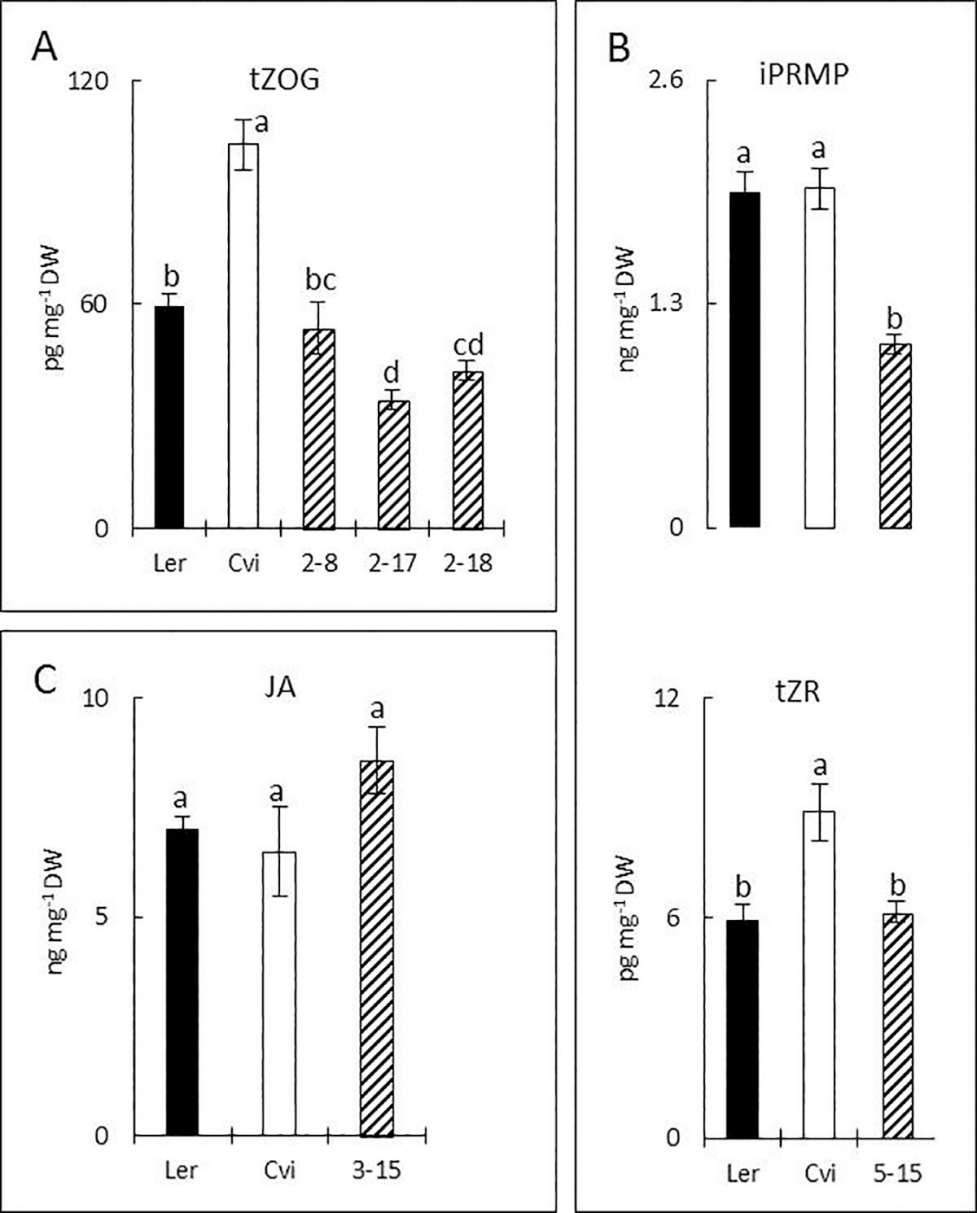
Confirmation of QTLs for tZOG, iPRMP, tZR and JA. (A), tZOG levels found in parental lines and three NILs, with Cvi-0 introgression in Ler-0 background (2–8, 2–17 and 2–18). For details, see maps of NILs in the supporting information (S1A Fig). (B), levels of iPRMP and tZR in NIL5-15 (S1B Fig). (C), JA levels in NIL3-15 (S1C Fig). Vertical bars present standard errors and letters on the top of each bar indicate significant differences between lines with a confidence interval of 95% (ANOVA, Duncan).

A large-effect QTL (11.7 LOD score) for the level of tZOG on chromosome 2 was confirmed using three NILs (S1A Fig). Significant lower levels of tZOG were observed in both NIL2-17 and NIL2-18, but not in NIL2-8 (Fig 5A). This might indicate that a responsible gene is located slightly below the above-described major QTL position, or that more than one locus is involved. In the overlapping introgression region of NIL2-17 and NIL2-18 several genes related to CK biosynthetic pathways are located (LOG2, LOG3, UGT73C1 and UGT73C5) [29, 30].

#### c. QTL for iPRMP and tZR at chromosome 5

A locus for levels of iPRMP and tZR on the bottom of chromosome 5 was evaluated using NIL5-15. The NIL showed significantly lower level of iPRMP than Ler, but the level of tZR was similar to that of Ler (Fig 5B, S1B Fig). These results suggest that a locus for the lower level of iPRMP is indeed located in the Cvi introgression region. While, a locus for the higher level of tZR may be located below CH.331L-Col because the introgression region for Cvi alleles in NIL5-15 does not fully cover the QTL region between CH.331L-Col and EG.205L. However, since these were only minor QTLs (2.5~3.0 LOD scores), the statistical power in the analyses might have been too low to detect allelic effects in the NILs. A known CK oxidase/dehydrogenase (CKX3) locates at the top of both QTL and the Cvi introgression in NIL5-15 [31].

#### d. QTL for JA at chromosome 3

The QTL for the level of JA at the bottom of chromosome 3 was evaluated with NIL3-15 (S1C Fig). The level of JA in NIL3-15 was higher than that in Ler, although there was no significant difference (Fig 5C). Since the region of Cvi introgression in NIL3-15 fully covers the significant interval of the QTL, a locus for the higher level of JA in Cvi can be located in between AD.182C and BH.109L-Col.

### Effects of loss-of-function of *UGT76C2* on CK metabolites and root phenotypic traits

To further elucidate genes affecting levels of CKs (tZRMP, tZ7G and tZ9G), we zoomed in on QTL regions at the top of chromosome 5. At this region (Fig 4A), containing QTLs for tZRMP, tZ7G, tZ9G and some of CK metabolite-ratios, several known CK genes are situated: two *LOGs* (*LOG6* and *LOG7*) and two *UGTs* (*UGT76C2* and *UGT76C1*) [32]. Lonely Guy (LOG) converts cytokinin nucleoside 5´-monophosphates (e.g., iPRMP and tZRMP) to free-bases (e.g., iP and tZ) [33]. Biologically active free bases are deactivated by *N-*glucosyltransferase, resulting in e.g., tZ7G [29, 30]. All these biosynthetic genes are supposed to be directly responsible for determining concentrations of tZRMP, tZ7G and tZ9G detected in the present QTL analysis. Thus, we chose LOG7 gene (At5g06300) and two zeatin *N*-glucosyltransferase genes (At5g05860 for UGT76C2 and At5g05870 for UGT76C1) that are known to be clearly expressed in roots of young seedling stages, as genetic components for further KO analyses [34].

**Fig 6:**
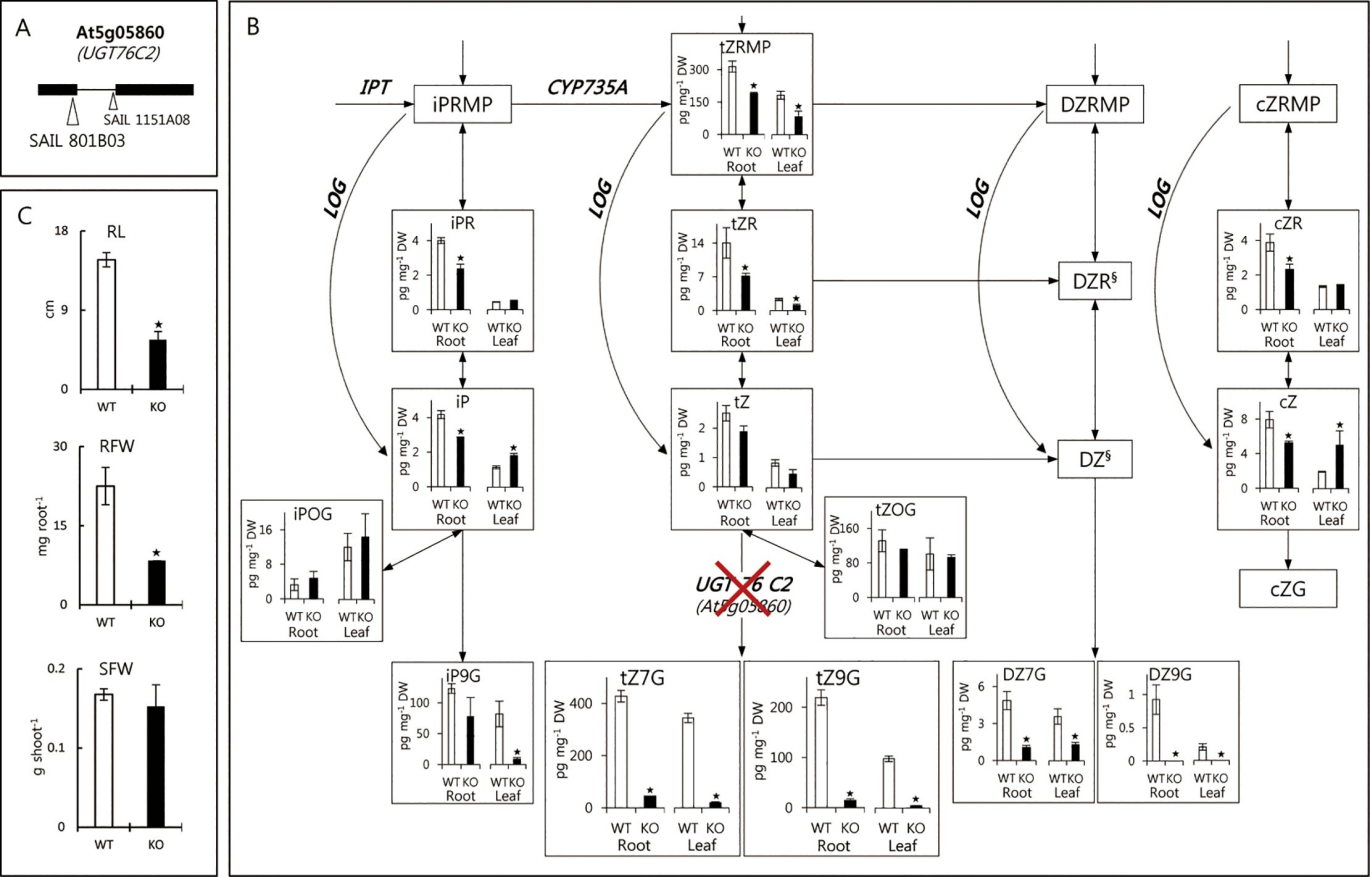
Changes of CK metabolite levels and phenotypic traits in the KO plant of UGT76C2 gene. (A), T-DNA insertions of two KO lines for the UGT76C2 gene (At5g05860); horizontal black bars and a line between indicate exons and an intron respectively. Triangles indicate position of insertions. (B), levels of CK metabolites in the wild type (Columbia-0) and the KO plant (SAIL 801B03). (C), phenotypic traits of root and shoot in the wild type and the KO plant. Stars on the top of graphs indicate significant differences in comparison with the wild type (*t-*test, P<0.01, five replicates for each line). Data are presented in a scheme of CK biosynthetic pathways. Significant differences of traits in the KO plant, as compared to the wild type are showed by star markers (t-test, P<0.01) Compounds in boxes without graph, e.g., iPRMP, were not included in the analysis. Metabolites superscripted with ^§^ were included in the analyses, but were under the limit of detection.

Among the tested KO lines for these genes, we obtained a homozygous T-DNA insertion line (SAIL 801B03) of UGT76C2 gene (Fig 6A, S2 Fig), which is known to be higher expressed in roots during the vegetative stage, as compared to *UGT76C1* [35]. For genes of LOG7 and UGT76C1, no homozygous T-DNA insertion line was obtained from the given KO lines.

Fig 6B illustrates part of the CK biosynthetic pathways and the changes of metabolic contents between the wild type and the KO plant. The KO plant of *UGT76C2* showed dramatic decreases of concentrations of tZ7G and tZ9G in roots. Levels of other CK *N-*glucosides (iP9G, DZ7G and DZ9G) from different side chains also decreased, which may be direct consequences of the loss-of-function of the gene. However, levels of CK *O*-glucosides (iPOG and tZOG) in both roots and leaves were hardly changed, showing *N-*glucosylation specificity of UGT76C2. The knocking-out of the gene also affected a wide range of levels of other CK metabolites. In roots, levels of the various metabolites (iPR, iP, tZRMP, tZR, tZ7G, tZ9G, DZ7G, DZ9G, cZR and cZG) decreased, whereas in leaves also opposite effects were found (iP and cZ). For this KO plant, significant morphological changes in roots were also observed. RL and RFW decreased by more than 60% in the KO plant compared to the wild type (Fig 6C), while only 9% reduction of SFW was observed.

## Discussion

### Hormone quantities and their correlations

A wide range of concentrations of hormones and their metabolites were found in the RILs. Except for ABA and cZR, concentration differences were several ten-fold between extremes in the segregating population, which was in strong contrast to the very limited variations of hormone levels found in 23-day-old roots of 13 Arabidopsis natural accessions in our previous study (e.g., iP and tZ, < 1.5 fold differences) [16]. Transgressive segregation has been found in many studies using bi-parent populations [21, 36, 37]. The most likely explanation for this phenomenon is the presence of both positive and negative alleles in each of the parental lines. In the RILs new allelic combinations will occur, leading to more extreme phenotypes. The limited natural variation, as observed in the accessions, points to natural selection leading to less extreme phenotypes.

Positive correlations between levels of tZ *N-*glucosides and RFW are similar to our previous results on natural accessions (r^2^ = 0.48 ~ 0.57) [16]. These are in agreement with the findings of Novak *et al*. [38] showing that the reduction of CK *N-*glucoside levels caused by CK oxidase/dehydrogenase (CKX7) overexpression resulted in the reduction of primary root elongation and lateral root growth. In the present study, similar morphological changes in root development were found in the KO plant of CK *N-*glucosyltransferase, proving a significant role of CK *N*-glucoside in root growth and development (Fig 6C). The underlying mechanism on how CK glucosides play a role in promoting root growth is unknown, but it has been used for exogenous application as a sugar-conjugate plant growth regulator [39]. The significant role of CK *N-*glucoside in plant productivity suggests that the regulation of CK glucoside contents can be a biochemical target in breeding programs, particularly for root systems.

Significant correlations between hormone compounds may point to common molecular mechanisms in their metabolism. It is noteworthy that a high correlation (r^2^ = 0.83) between tZ7G and tZ9G was observed. The average concentration ratio (peak area comparison in chromatograms) observed in the whole RILs was 0.97 : 1 (tZ7G : tZ9G). The transgressive segregation of this trait (Fig 1) suggests that the ratio may be a consequence of polygenic inheritance. Hou *et al*. [29] suggested that the abundance of tZ7G compared to tZ9G may result from the specificity of CK *N*-glucosyltransferases (thus a biological reaction), but the amount of tZ9G may also be the result of tautomerism between two constitutional isomers in aqueous solution (non-biological reaction). But Leon *et al*. [40] estimated that *N9*-glucosylation of tZ is rather stable in relation with entropic contribution (*Δ*G°), concluding that approximately equal or lower amounts of *N7*-isomer should be present at room temperature. In the present study, the existence of at least two independent QTLs for tZ9G level provides empirical evidence that the quantitative genetic component is involved in determining the trait (Fig 2), implying that an unknown enzymatic pathway (isomerization) might be involved in the conversion of two CK *N-*glucoside isomers.

### QTLs for hormone metabolites and their ratio traits

The total number of significant QTLs for 15 metabolites in five groups of hormones was 14. The average number of QTL per hormone metabolite was strikingly lower compared to those of diverse growth-related traits, mineral contents, or seed traits as reported in previous studies using the same RIL population [20, 37, 41–43]. Except for the two loci for tZOG, tZ7G and tZ9G, most QTLs explained less than 20% of phenotypic variance, suggesting that other minor-effect loci remained undetected. It is also likely that hormone variations found in the RILs are partly contributed by non-genetic factors, e.g. environmental perturbation, despite all attempts to control and standardize the growth conditions of the plants.

Another reason to explain the relative low number of hormone QTLs is differential spatial distributions of hormone metabolites in distinct root tissues and/or cell types. The described auxin maximum in the root apex indicates that local biosynthesis and polar transport results in auxin gradients and differential distribution [44]. Antoniadi *et al*. [45] reported that a concentration gradient and cell-type-specific distribution for CKs are present in the Arabidopsis root apex. Since hormone quantification in our study was determined from homogeneous powders of the whole root system, it is impossible to draw conclusions on differential hormone concentrations in different cell types. Such mean effects present in the root samples could influence QTL numbers and their relative contribution on LOD scores for hormones and their metabolites. It can also explain why no QTLs for levels of auxin and ABA were above the significant threshold (data not included). A way to avoid this problem—local dissection of the complex root system before extraction—would have also brought about heterogeneity of sampling due to unclear boundaries in the continuous meristem-elongation-differentiation-transition zones, and the sensitivity of the detection method might have been too low, and hence why it was not done in this study.

The co-localization of QTLs for metabolite-ratios with those for single compound suggests that the underpinning gene(s) can be involved in the metabolism of either early or late pathways (e.g., at chromosome 2 and 5 in Fig 2). In addition, a newly observed QTL for the ratio between tZ7G and tZ9G in chromosome 5 would be another indication that the metabolic conversion of these tZ *N*-glucosides is controlled genetically, differing with the previous assertion that it resulted from either non-biological reaction or enzymatic *N*-glucosylation [29].

### Detection of developmental-specific QTLs from vegetative and flowering lines

The difference of QTL profiles between vegetative and flowering sub-populations suggests that some QTLs related to hormone levels in roots are development-specific (Fig 3). The transition to flowering can trigger changes of source-sink relationships in leaves through hormone signalling (e.g., gibberellins in Arabidopsis) [46, 47]. Our finding of differential QTL profiles for two distinct development stages implies that the transition to flowering affects root traits and hormone contents.

Using rather mature plants provides advantages to analyse the flowering-specific loci that would not have been possible in seedlings. QTLs for specific developmental stages have been also identified in other studies, e.g., for linolenic acid content in soybean seed, plant height in Brassica and fruit size in cucumber [48–50]. All these studies used whole RIL populations repetitively for different developmental stages, and then a QTL for each group was re-analysed separately. Our approach can be applied to find whether an interesting QTL is temporally functional at a particular stage, especially in roots, that do not show such obvious developmental changes as seen in shoots. In the present study, numbers of significant QLTs for the three categories were 8 : 5 : 2 (vegetative : flowering : non-development-specific or constant). It implies that a large number of loci for root growth and hormone levels are temporally functional rather than being continuously active for the two developmental stages.

In the vegetative lines, two QTLs were newly found that were detected neither in the whole populations, nor in the flowering lines, viz., for SA and RL. Thus, these loci are only (or mainly) active in the vegetative stage, and including data from flowering plants only adds non-genetic variation, thus obscuring the QTLs. RFW has a vegetative- and a flowering-specific QTL, but RL only has a vegetative-specific QTL, suggesting that at the flowering stage root weight is still regulated by other genetic factor, or triggered by signalling from upper parts of the plant.

### Pleiotropic effects of UGT76C2 gene on CK metabolism and different roles of CK glucosides in roots and leaves

Since the two *trans*-zeatin N-glucosyltransferase genes (At5g05860 and At5g05870) both closely positioned to BH-144L on chromosome 5 where LOD scores of the QTL regions were highest, it is likely that one of these genes (or both) are responsible for determining levels of tZ7G and tZ9G. However, due to lack of detailed makers in the region between the end-point of Cvi introgression in NIL5-1 and the start-point of Cvi introgression in NIL5-2, it was uncertain which of the two genes caused (most of) the effect.

Pleotropic effects of UGT76C2 gene, encoding an *N*-glucosyltransferase, on many CK metabolites were found in the present study. In Arabidopsis roots, the loss-of-function of this gene greatly reduced levels of not only *N-*glucosides, but also other upstream metabolites in both tZ pathway and other side (lateral) chains in CK metabolism. In leaves, contents of CK metabolites were less affected, except compounds in the tZ pathway, as also found in a recent study [35]. Wang *et al*. [51] also elucidated the functional role of *UGT76C2* in Arabidopsis seedling (2 weeks) using KO plants and overexpressors. They reported significant reductions of levels of CK *N-*glucosides but little changes of other CK metabolites. In comparison with the findings of Wang *et al*. [51], the observation of more extensive changes of the contents of CK metabolites in the present study may be caused by differences of organs (only roots in the present study) and age (5 weeks). Unlike CK *N-*glucosides, levels of *O-*glucosides in the KO plants hardly changed in both studies. This is consistent with another study that two *N-*glucosyltransferases (*UGT76C2* and *UGT76C1*) in Arabidopsis are involved in CK *N-*glucosylation, and three other UGTs are separately responsible for *O-*glucosylation [29]. The reduction of CK *N-*glucoside levels resulted in significant dwarfism of root phenotypes in our study. This is consistent with the findings of Kollmer *et al*. [38] showing that levels of CK *N-*glucosides positively correlated with root growth and development. However, it is difficult to evaluate to what extent the changes of levels of other CK metabolites directly affected root growth.

The decrease of levels of *N*-glucosides in both roots and leaves is in agreement with transcriptomic data studied by Schmid *et al*. [52], showing that the At5g05860 gene, coding for UGT76C2, is substantially transcribed in both roots and leaves at the early vegetative stage, but becomes inactive in roots during flowering. The reduction of levels of CK *N*-glucosides in leaves hardly affected shoot weight and other aerial phenotypes. This is consistent with vegetative-specificity of the QTLs for levels of tZ7G and tZ9G, although it is hard to conclude whether only *UGT76C2* is responsible for the QTL or if other genes are also involved. Based on presented data, it is likely that CK *N-*glucosides have different physiological roles in roots compared to shoots since depletion of *N*-glucosides only significantly affected root growth (Fig 6C).

## Conclusion

In conclusion, QTL analysis based on hormone levels in Arabidopsis roots revealed genetic loci involved in regulation and/or metabolism of CK. Flowering has a profound effect on many of the QTLs detected. Differential regulations of hormone QTLs in the vegetative vs. flowering stages suggest that it may be interesting to study other RIL populations derived from late-flowering lines, in which all progenies can be in the vegetative stage at the sampling time. For a better understanding of hormone regulation at the whole-plant level, hormone-metabolic QTL studies should be extended to other organs, e.g., leaves. The loss-of-function analysis of *UGT76C2*, a gene underlying a major QTL for levels of tZ7G and tZ9G suggests that CK *N*-glucosides play an important role in root development.

## Supporting information

S1 Table Summary of multiple reaction monitor (MRM) transitions used for hormone quantification in UPLC-ESI-triquadrupole mass spectrometer. (DOCX)

S2 Table Significant QTLs for hormone levels and root phenotypic traits. ^§^ indicates loci for traits observed by QTL analysis using only vegetative lines.
(DOCX)

S1 Fig Maps of NILs (QTL confirmation study: Case-2, 3 and 4). (A), genotypes of NIL 2–8, 2–17 and 2–18 for tZOG-QTL. The vertically dotted graph on the right shows significant QTL region wide covering 35.6 cM distance, from BF.221L to DF.140C. Black columns indicate QTL regions in 2-LOD confidence interval. The nearest marker at the highest LOD score was Erecta/GPA1. NIL2-8 contains an additional introgression of Cvi at BF.221L, where locates up ward out of QTL region. For NIL2-18, two interruptions with Ler alleles were found in the Cvi introgression. Several uncharacterized UDP-glucosyltransferase genes (*UGTs*) including zeatin *UGTs* (*UGT73C1* and *UGT73C5*) and *LOG2* situate in between HH.320L-Col and DF.140C. (B), genotype of NIL 5–15 for QTLs of iPRMP and tZR. (C), genotype of NIL3-15 for JA-QTL.
(TIFF)

S2 Fig Homozygosity test of two KO lines (SALL 801B03 and SAIL 1151A08). The genotyping of T-DNA insertion was based on a comparison between two PCR reactions: a set of the forward and the reverse primer in the gene, F/R; a set of Bp (BPos in T-DNA) and the reverse primer of the gene, B/R. For SAIL 801B03, Line 4, Line5 and Line 6 were heterozygous, but Line 7 was homozygous, which was chosen for the comparion test between the wild type and the KO plant (Fig 6B). For SAIL 1151A08, Line 6 was homozygous, but significant changes of levels of tZ *N*-glucosides were not observed (S3 Fig) because T-DNA insertion might be taken place in the intron region (Fig 6A).
(TIF)

S3 Fig Levels of tZ7G and tZ9G in two KO lines for At5g05860. (TIF)
