# Supplementary material for: Quantitative trait loci analysis of hormone levels in Arabidopsis roots
Source: PLoS One. 2019 Jun 28;14(6):e0219008. doi: 10.1371/journal.pone.0219008 (PMC6599112; doi:10.1371/journal.pone.0219008)
Supplement: S1 Table — (DOCX) [file pone.0219008.s001.docx]

S1 Table

| Analytes | UPLC  Gradient | RT | ESI | MRM  Function | Transition | Cone voltage | Collision energy | Internal standard | RT | Transition | Cone voltage | Collision energy |
| --- | --- | --- | --- | --- | --- | --- | --- | --- | --- | --- | --- | --- |
| tZ | 1 | 3.78 | + | 2 | 220.1>136.1 | 20 | 18 | [^2^H_6_] tZ | 3.75 | 225.1>136.9 | 20 | 18 |
| tZR | 1 | 5.04 | + | 3 | 352.1>136.1 | 28 | 20 | [^2^H_5_] tZR | 5.01 | 357.1>136.9 | 28 | 20 |
| iP | 1 | 6.15 | + | 1 | 204.1>136.1 | 20 | 18 | [^2^H_6_] iP | 6.11 | 210.1>137.1 | 20 | 18 |
| iPR | 1 | 7.60 | + | 1 | 336.1>204.1 | 28 | 18 | [^2^H_6_] iPR | 7.55 | 342.2>210.2 | 28 | 18 |
| cZ | 1 | 4.01 | + | 2 | 220.1>136.1 | 20 | 18 | [^2^H_6_] tZ | 3.75 | 225.1>136.9 | 20 | 18 |
| cZR | 1 | 5.31 | + | 3 | 352.1>136.1 | 28 | 16 | [^2^H_5_] tZR | 5.01 | 357.1>136.9 | 28 | 20 |
| tZOG | 1 | 3.84 | + | 2 | 382.1>220.1 | 30 | 16 | [^2^H_5_] tZ9G | 4.02 | 387.2>225.2 | 30 | 20 |
| tZ7G | 1 | 3.86 | + | 2 | 382.1>220.1 | 30 | 20 | [^2^H_5_] tZ9G | 4.02 | 387.2>225.2 | 30 | 20 |
| tZ9G | 1 | 4.04 | + | 2 | 382.1>220.1 | 30 | 20 | [^2^H_5_] tZ9G | 4.02 | 387.2>225.2 | 30 | 20 |
| DZ | 1 | 3.95 | + | 2 | 222.1>136.1 | 20 | 18 | [^2^H_3_] DZ | 3.91 | 225.1>136.1 | 20 | 18 |
| DZR | 1 | 5.10 | + | 3 | 354.1>136.1 | 28 | 22 | [^2^H_3_] DZR | 5.06 | 357.2>149.1 | 28 | 22 |
| DZOG | 1 | 4.18 | + | 2 | 384.1>222.1 | 30 | 16 | [^2^H_5_] tZ9G | 4.02 | 387.2>225.2 | 30 | 20 |
| DZ7G | 1 | 4.07 | + | 2 | 384.1>136.1 | 30 | 20 | [^2^H_5_] tZ9G | 4.02 | 387.2>225.2 | 30 | 20 |
| DZ9G | 1 | 4.17 | + | 2 | 384.1>136.1 | 30 | 20 | [^2^H_5_] tZ9G | 4.02 | 387.2>225.2 | 30 | 20 |
| tZRMP | 1 | 3.58 | + | 2 | 432.1>220.1 | 34 | 22 |  |  |  |  |  |
| IAA | 2 | 4.15 | + | 1 | 176.1>130.1 | 18 | 16 | [^13^C_6_] IAA | 4.14 | 182.1>136.1 | 18 | 16 |
| OxIAA | 2 | 3.62 | + | 1 | 191.9>145.9 | 18 | 16 |  |  |  |  |  |
| ABA | 2 | 5.28 | + | 2 | 265.2>247.2 | 18 | 14 | [^2^H_6_] ABA | 5.26 | 271.2>253.2 | 18 | 14 |
| JA | 2 | 6.00 | + | 3 | 211.2>133.1 | 18 | 16 | [^2^H_4_] JA | 5.98 | 216.2>135.3 | 18 | 16 |
| SA | 2 | 4.76 | - | 2 | 136.9>92.9 | 25 | 15 | [^2^H_4_] SA | 4.75 | 140.9>96.9 | 25 | 15 |
