# Supplementary material for: Quantitative trait loci analysis of hormone levels in Arabidopsis roots
Source: PLoS One. 2019 Jun 28;14(6):e0219008. doi: 10.1371/journal.pone.0219008 (PMC6599112; doi:10.1371/journal.pone.0219008)
Supplement: S2 Table — § indicates loci for traits observed by QTL analysis using only vegetative lines. (DOCX) [file pone.0219008.s002.docx]

S2 Table

|  | Trait | Chrom-osome | | cM  interval | | LOD (MQM) | | Locus  region | Nearest  marker | Explained (%), MQM | Additive  effect |  |
| --- | --- | --- | --- | --- | --- | --- | --- | --- | --- | --- | --- | --- |
| Hormone compounds | iPRMP | 5 | | 94.9~117 | | 2.5 | | GB.102L-Col/105C~HH.122C/120L | DF.199L | 8 | + |  |
|  | tZRMP | 5 | | 2~14.8 | | 6.6 | | FD.207L~EC.198L-Col | CH.690C | 16 | - |  |
|  | tZR | 5 | | 95.9~116 | | 2.7 | | GB.102L-Col/105C~HH.122C/120L | DF.119L | 8 | - |  |
|  | tZOG | 2 | | 45.6~51.2 | | 11.7 | | FD.150C~GD.298C | Erecta | 24 | + |  |
|  |  | 3 | | 0~5.8 | | 4.1 | | DF.77C~EG.75L | DF.77C | 7 | - |  |
|  |  | 4 | | 39.6~80.2 | | 3.1 | | CD.84C-Col/85L~BH.342C/DHS1 | CH.70L/71C-Col | 5 | - |  |
|  | tZ7G | 5 | | 1~11.6 | | 8.0 | | FD.207L~EC.198L-Col | BH.144L | 20 | + |  |
|  | tZ9G | 1 | | 0~30.9 | | 2.9 | | PVV4~CC.98L-CH.160L-Col | PVV4 | 6 | - |  |
|  |  | 3 | | 0~5.8 | | 5.2 | | DF.77C~EG.75L | DF.77C | 11 | - |  |
|  |  | 5 | | 5.3~10.6 | | 11.0 | | CH.690C~EC.198L-Col | BH.144L | 25 | + |  |
|  | DZOG | 1 | | 16.3~46.7 | | 2.9 | | EC.480C~GB.112L | CH.160L-Col | 7 | - |  |
|  |  | 1 | | 52.1~67 | | 3.3 | | GB.112L~CD.89C | GD.97L | 9 | + |  |
|  | JA | 3 | | 69.3~85.8 | | 3.3 | | FD.98C~HH.90L-Col | HH.171C-Col/173L | 9 | - |  |
|  | SA | 1**^§^** | | 35.7~56.1 | | 3.6 | | CC.98L-Col/101C~GD.97L | AD.106L-Col | 20 | + |  |
| Ratios (precursor-to- product) | iPRMP/tZRMP | 5 | | 0~10.6 | | 5.9 | | FD.207L~EC.198L-Col | BH.144L | 17 | + |  |
|  | tZRMP/tZR | 5 | | 2~33.1 | | 3.6 | | FD.207L~DF.184L-Col | EC.198L-Col | 10 | - |  |
|  | tZ/tZOG | 2 | | 38.4~51.2 | | 6.3 | | FD.85C~GD.298C | Erecta | 16 | - |  |
|  | tZ/tZ7G | 5 | | 0~15.8 | | 3.0 | | FD.207L~ BH.107L-Col | BH.144L | 8 | - |  |
|  | tZ/tZ9G | 5 | | 0~15.8 | | 3.0 | | FD.207L~ BH.107L-Col | BH.144L | 9 | - |  |
|  | tZ7G/tZ9G | 5 | | 62.5~81.5 | | 3.3 | | BH.96L-Col~AD.75C-Col | CD.116L | 10 | - |  |
|  | tZOG/DZOG | 2 | | 44.6~51.2 | | 7.0 | | FD.85C~GD.298C | Erecta | 19 | + |  |
| Phenotypic  traits | RL | 5**^§^** | | 9.6~36.8 | | 3.2 | | BH.144L~GH.117C | BH.107L-Col | 16 | - |  |
|  | RFW | | 1 | | 2~15.1 | | 7.3 | PVV4~EC.480C | HH.335C-Col/PhyA | 16 | - | |
|  |  |  | 5 | | 93~117 | | 4.3 | CC.262C~HH.122C/120L | DF.119L | 10 | + | |
|  | SFW | | 1 | | 0~27.3 | | 5.6 | PVV4~CH.160L-Col | PVV4 | 14 | - | |
|  |  |  | 2 | | 61.2~70.8 | | 3.2 | BH.120L-Col~EC.235L-Col | EC.235L-Col | 7 | + | |
|  | Flowering, stalk number | | 1 | | 0~10 | | 15.8 | PVV4~HH.335C-Col/PhyA | PVV4 | 34 | - | |
