# Supplementary figures and images for: Quantitative trait loci analysis of hormone levels in Arabidopsis roots
Source: PLoS One. 2019 Jun 28;14(6):e0219008. doi: 10.1371/journal.pone.0219008 (PMC6599112; doi:10.1371/journal.pone.0219008)

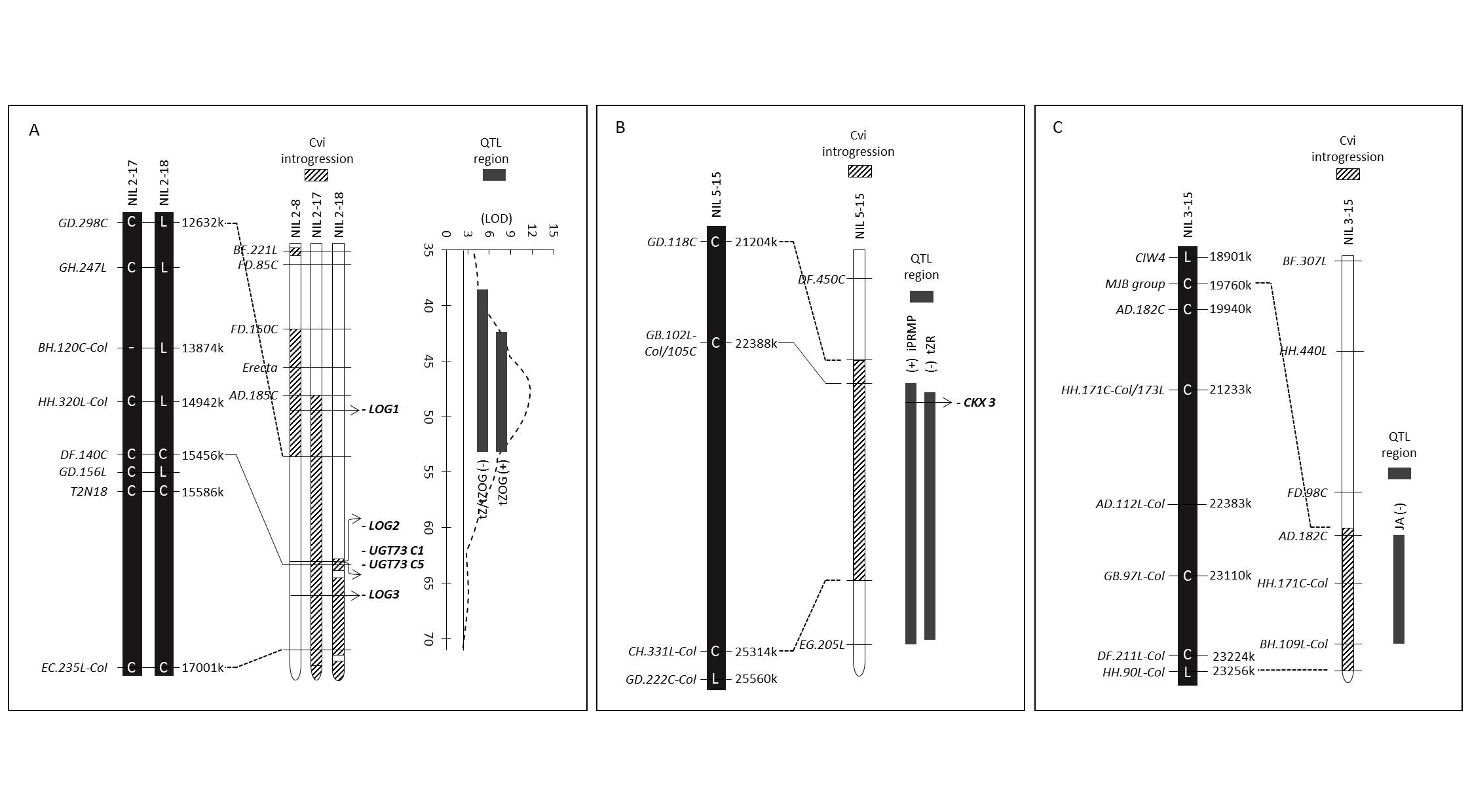

Supplement: S1 Fig — (A), genotypes of NIL 2–8, 2–17 and 2–18 for tZOG-QTL. The vertically dotted graph on the right shows significant QTL region wide covering 35.6 cM distance, from BF.221L to DF.140C. Black columns indicate QTL regions in 2-LOD confidence interval. The nearest marker at the highest LOD score was Erecta/GPA1. NIL2-8 contains an additional introgression of Cvi at BF.221L, where locates up ward out of QTL region. For NIL2-18, two interruptions with Ler alleles were found in the Cvi introgression. Several uncharacterized UDP-glucosyltransferase genes (UGTs) including zeatin UGTs (UGT73C1 and UGT73C5) and LOG2 situate in between HH.320L-Col and DF.140C. (B), genotype of NIL 5–15 for QTLs of iPRMP and tZR. (C), genotype of NIL3-15 for JA-QTL. (TIFF) [file pone.0219008.s003.tiff]

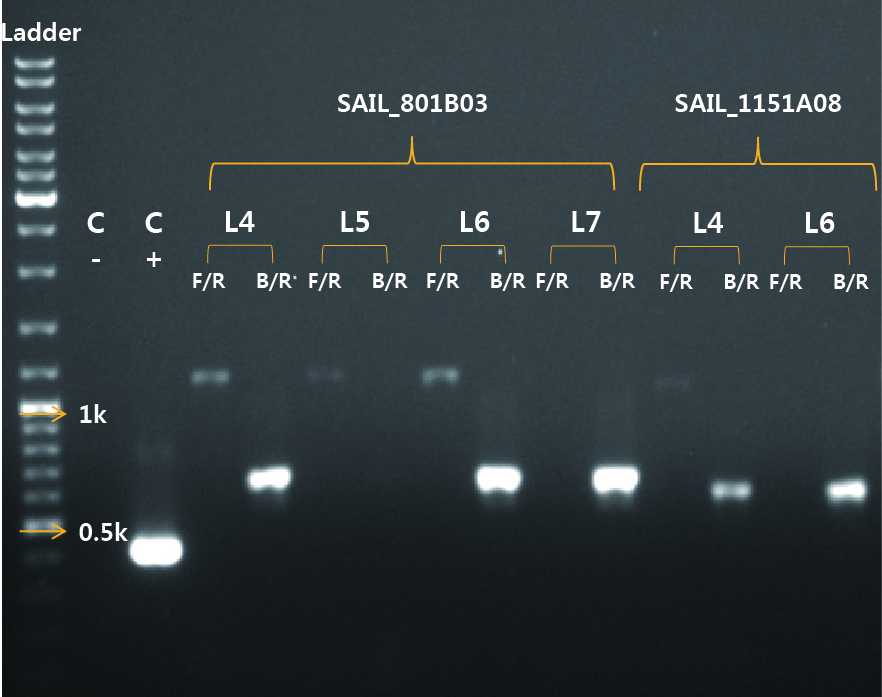

Supplement: S2 Fig — The genotyping of T-DNA insertion was based on a comparison between two PCR reactions: a set of the forward and the reverse primer in the gene, F/R; a set of Bp (BPos in T-DNA) and the reverse primer of the gene, B/R. For SAIL 801B03, Line 4, Line5 and Line 6 were heterozygous, but Line 7 was homozygous, which was chosen for the comparion test between the wild type and the KO plant (Fig 6B). For SAIL 1151A08, Line 6 was homozygous, but significant changes of levels of tZ N-glucosides were not observed (S3 Fig) because T-DNA insertion might be taken place in the intron region (Fig 6A). (TIF) [file pone.0219008.s004.tif]

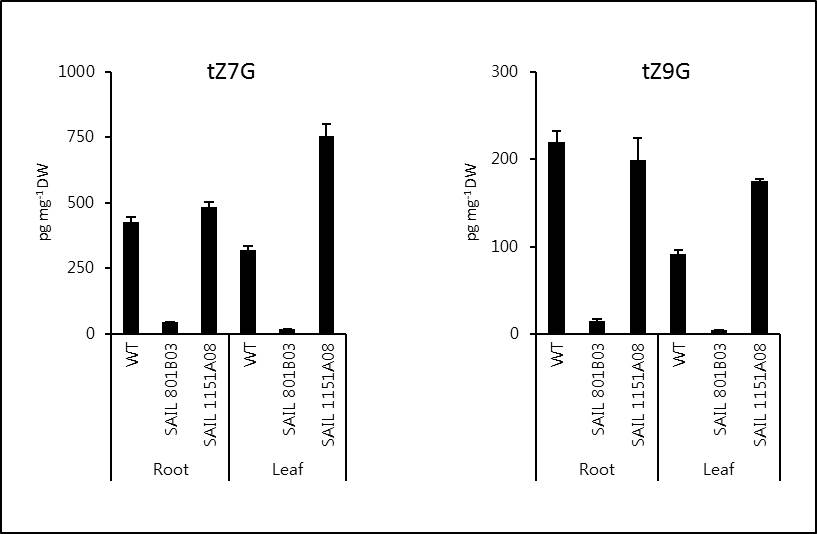

Supplement: S3 Fig — (TIF) [file pone.0219008.s005.tif]
